# Impact of domiciliary administration of NSAIDs on COVID-19 hospital outcomes: an unCoVer analysis

**DOI:** 10.3389/fphar.2023.1252800

**Published:** 2023-10-09

**Authors:** Elena Salvador, Cristina Mazzi, Nicoletta De Santis, Giulia Bertoli, Antonija Jonjić, Miran Coklo, Marek Majdan, José L. Peñalvo, Dora Buonfrate

**Affiliations:** ^1^ Department of Infectious Tropical Diseases and Microbiology, IRCCS Sacro Cuore Don Calabria Hospital, Negrar, Verona, Italy; ^2^ Clinical Research Unit, IRCCS Sacro Cuore Don Calabria Hospital, Negrar, Verona, Italy; ^3^ Centre for Applied Bioanthropology, Institute for Anthropological Research, Zagreb, Croatia; ^4^ Institute for Global Health and Epidemiology, Trnava University, Trnava, Slovakia; ^5^ Unit of Non-Communicable Diseases, Institute of Tropical Medicine, Antwerp, Belgium; ^6^ Global Health Institute, University of Antwerp, Antwerp, Belgium

**Keywords:** COVID-19, SARS-CoV-2, outcome, treatment, NSAIDs, uncover

## Abstract

**Background:** Effective domiciliary treatment can be useful in the early phase of COVID-19 to limit disease progression, and pressure on hospitals. There are discrepant data on the use of non-steroidal anti-inflammatory drugs (NSAIDs). Aim of this study is to evaluate whether the clinical outcome of patients who were hospitalized for COVID-19 is influenced by domiciliary treatment with NSAIDs. Secondary objective was to explore the association between other patient characteristics/therapies and outcome.

**Methods:** A large dataset of COVID-19 patients was created in the context of a European Union-funded project (unCoVer). The primary outcome was explored using a study level random effects meta-analysis for binary (multivariate logistic regression models) outcomes adjusted for selected factors, including demographics and other comorbidities.

**Results:** 218 out of 1,144 patients reported use of NSAIDs before admission. No association between NSAIDs use and clinical outcome was found (unadj. OR: 0.96, 95%CI: 0.68-1.38). The model showed an independent upward risk of death with increasing age (OR 1.06; 95% CI 1.05-1.07) and male sex (1.36; 95% CI 1.04-1.76).

**Conclusion:** In our study, the domiciliary use of NSAIDs did not show association with clinical outcome in patients hospitalized with COVID-19. Older ages and male sex were associated to an increased risk of death.

## 1 Introduction

Coronavirus disease 2019 (COVID-19) has represented a global health, social and economic challenge, overwhelming healthcare systems in many countries and heavily burdening others.

Healthcare workers have been struggling to provide care, sometimes in presence of limited bed and respirator capacity, in particular in intensive care units (Fagiuoli et al., 2020). Hence, the evaluation of treatments that, given at early stage preferably at home, could prevent the progression of the infection to severe disease, was deemed relevant (Perico et al., 2023).

Due to their analgesic, anti-inflammatory, and antipyretic effect (Kushner et al., 2022), in combination with wide availability and affordability, non-steroidal anti-inflammatory drugs (NSAIDs) became appealing for symptomatic treatment of COVID-19 in both outpatients and inpatients.

In mice models, the use of NSAIDs appeared to reduce the production of pro-inflammatory cytokines and neutralizing antibodies, supporting the role of NSAIDs for the humoral response to SARS-CoV-2 (Matute-Bello et al., 2008; Chen et al., 2021).

Studies unveiling the pathogenesis of COVID-19 highlighted the role of inflammation dysregulation for disease progression, with production of pro-inflammatory cytokines that activate neutrophils, causing pulmonary epithelium and endothelial damage (Merad et al., 2022). The abnormal systemic inflammatory response is thought to be at the basis of the disease progression, even after viral clearance (Merad et al., 2022).

Moreover, mononuclear inflammatory infiltrate with lymphocytes and macrophages in the lungs of patients with severe forms of COVID-19 confirms a significant role of the host innate immune system in the immunopathology of the disease (Gustine and Jones, 2021). These data have prompted the investigation of anti-inflammatory drugs able to prevent lung damage in COVID-19.

Since the early phases of the pandemic, corticosteroids (specifically dexamethasone) have been considered a milestone in the treatment of hospitalized COVID-19 patients receiving respiratory support (Peter et al., 2021). Studies showed that it is crucial to administer steroids during the early inflammatory phase (i.e., around the second week of illness) to reduce the risk of disease progression and death (Griffin, 2022).

While there is consensus on the role of steroids for the treatment of severe COVID-19, there are discrepant data about the use of NSAIDs in early phases of SARS-CoV-2 infection (Bhimraj et al).

Early during the pandemic, there was some concern about their use for treating COVID-19 symptoms because of a supposed increased risk of cardiovascular events (Little, 2020), as well as the possibility to exacerbate COVID-19 symptoms, via upregulation of angiotensin-converting enzyme 2 (ACE2) receptors in the lungs, arteries, heart, kidney, and intestines (Kuba et al., 2005). On the other hand, it was argued that, due to their anti-inflammatory properties, NSAIDs might mask inflammation and fever, thus delaying and complicating COVID-19 diagnosis (Kragholm et al., 2021). Studies were hence carried out to evaluate the safety of NSAIDs use during SARS-CoV-2 infection and a possible association with disease progression. Not only their safety was demonstrated (Drake et al., 2021), but further studies suggested NSAID’s crucial role for the management of outpatients with early symptoms of COVID-19, even with an impact on preventing severe disease progression (Perico et al., 2023). However, there is no definitive evidence on this (Zhao et al., 2022). Moreover, it is not clear whether early administration of NSAIDs may be useful to reduce the proportion of individuals needing hospitalization and respiratory support.

UnCoVer (Unravelling data for rapid evidence-based response to COVID-19) is a project funded by the European Commission within the Horizon 2020 framework, aiming to provide a research platform for the expert use of real-world data (RWD) originating from different countries within a privacy-preserving federated analytics platform (Peñalvo et al., 2021). The main strength of the platform is the large amount of patient-level data on COVID-19 cases provided by most of the 29 partner institutions participating on the project, which included both European Union (EU) and non-EU countries.

We benefited from the unCoVer platform while carrying out this study, whose primary objective was to evaluate whether domiciliary treatment with NSAIDs might be associated with a better outcome (lower mortality) in individuals admitted to hospital with COVID-19, compared to individuals who did not take NSAIDs before hospital admission.

Secondary objectives were: i) to evaluate the influence of age, sex and comorbidities on the outcome of the two main subgroups (patients taking or not NSAIDs at home); ii) to evaluate if domiciliary treatment with oral steroids and antibiotics might be associated with a better outcome (lower mortality) in individuals admitted to hospital with COVID-19, compared to individuals who did take other treatments before hospital admission.

## 2 Materials and methods

### 2.1 Study design and data sources

This was a multicentric observational study. All data present in the unCoVer platform, originating from patients hospitalized for COVID-19 from March 2020 to March 2023 and made available in Opal/DataSHIELD were deemed eligible. We included all patients for whom the data required for this study were available. The platform is available at: https://uncover-eu.net/data/.

### 2.2 Ethics requirements

The unCoVer project followed ethics and data protection rules in compliance with the European and each participant country’s laws, as assessed by a specific work package (WP2). The unCoVer study has been approved by the Institutional Review Board of the Institute of Tropical Medicine in Antwerp (IRB/RR/ac/151, protocol number 1524/21), coordinating site, and by the pertinent Ethics Committees of the participating Institutions. Partner institutions guaranteed the acquisition of informed consent locally.

### 2.3 Statistical analysis

DataSHIELD-R packages (R software, version 4.2.3) were used for all analyses (Marcon et al., 2021). Descriptive statistics included number of patients, median and interquartile range (IQR) for continuous variables, and frequency and percentage for categorical variables. Each outcome was reported along with its 95% confidence interval (CI). The association between home medication with NSAIDs or other home medication (steroids/antibiotics/ACE inhibitors) and hospital outcome was explored using a study level random effects meta-analysis for binary (multivariate logistic regression models) outcomes adjusted for selected factors, including demographics (e.g., age, sex) and the presence of one, two or more comorbidities (e.g., obesity, chronic obstructive pulmonary disease, malignancies, cardiovascular disease, diabetes, chronic kidney or liver disease, rheumatism). Random effects under restricted maximum likelihood (REML) were used to pool the model’s results.

## 3 Results

The analyses were conducted for a total of 1,273 patients, available in two databases located in Croatia (*N* = 202) and Slovakia (*N* = 1,071). All other datasets missed at least one of the necessary variables for the analysis (e.g., domiciliary treatment). Overall, 54.1% individuals were female, and median age was 56 years (IQR 45-64). Information about domiciliary use of NSAIDs was available for 1,144 patients, of whom 218 (19.1%) reported use before admission. Of the 218 patients using NSAIDs, 85 (39.0%) died during hospitalization. Oral steroids were used by 103 (8.1%), antibiotics by 240 (18.9%), and ACE inhibitors by 401 (31.5%) of the patients. At least one comorbidity was present in 738 (58.0%) of the enrolled patients.

NSAIDs use before hospital admission was not associated with patients’ outcome during hospitalization (unadj. OR: 0.96, 95%CI: 0.68-1.38).

Underlying comorbidities or additional domiciliary therapies did not influence the outcome. Increasing age and male sex were independently associated with an upward risk of death: OR 1.06 (95% CI 1.05-1.07), and 1.36 (95% CI 1.04-1.76), respectively. Table 1 shows the results of the model.

**TABLE 1:** Association of demographics, underlying conditions, and domiciliary treatment with patient in-hospital death.

	Pooled estimate	
Parameter	OR	95% CI
Comorbidity, 1 (Ref. 0)	0.64	0.24–1.72
Comorbidity, 2 or more (Ref. 0)	1.23	0.89–1.70
Age, ys	1.06	1.05–1.07
Gender, Male (Ref. Female)	1.36	1.04–1.76
Antibiotic use, Yes (Ref. No)	0.79	0.57–1.11
ACE inhibitors use, Yes (Ref. No)	0.95	0.72–1.25
NSAIDs use, Yes (Ref. No)	1.11	0.74–1.65
Oral steroids use, Yes (Ref. No)	1.11	0.68–1.77

OR, odds ratio; CI, confidence intervals; ACE, Angiotensin-converting enzyme; NSAIDs, Non-steroidal anti-inflammatory drugs.

## 4 Discussion

In our study, domiciliary treatment with either NSAIDs or other considered drugs (oral steroids and antibiotics) did not influence the patient clinical outcome. An increased risk of death was instead associated with older age and male sex.

Our primary outcome is hence in line with the results of previous studies showing no association between domiciliary use of NSAIDs and clinical outcome in people hospitalized for COVID-19 (Drake et al., 2021). While other studies demonstrated some benefit from early use of NSAIDs (Perico et al., 2023), the data are not entirely discrepant with our findings, as some works focused on different outcomes, such as reduced need for hospitalization. Moreover, here we considered the NSAIDs altogether, while there might be some differences between different drugs of this class (Li et al., 2023). For instance, in a retrospective analysis by Gordon et al., the use of indomethacin, but not of celecoxib, by COVID-19 outpatients was related to a reduced risk of hospitalization (Gordon et al., 2020).

We should consider also that hyper-inflammation is not the only factor contributing to disease severity and mortality in COVID-19 infected patients. A recently published study shows that patients admitted to the intensive care unit with a high release of viral components to the circulation (“viral storm”) have higher mortality than those with lower viral load (Bermejo-Martin et al., 2023). Indeed, antiviral drugs and monoclonal antibodies demonstrated to reduce the risk of hospitalization and death when given during the first days since symptoms onset (Akinosoglou et al., 2023; Saravolatz et al., 2023). This was at least true for the first viral variants, as the study by Bermejo-Martin et al. was conducted during the first year of emergence of COVID-19, and monoclonal antibodies demonstrated less effective for variants that developed afterwards (Planas et al., 2021; Takashita et al., 2022).

All along the pandemic, we saw that emerging viral variants could lead to different clinical presentations and response to treatment (Akkız, 2022). Although randomized controlled trials are the best studies to collect evidence on drug efficacy, observational studies are also useful. They permit to monitor the effectiveness of proposed treatments and detect changes in clinical manifestations, which may prompt the need for randomized controlled trials. The availability of large datasets from different geographical sites strengthens this opportunity, giving an overview of the impact of the infection at individual and public health levels, in a broader context (Tacconelli et al., 2022). The unCoVer platform was set up with this scope and, although data from only two participating sites could be used for the present study, demonstrated that it is possible to share and analyze data collected in different datasets for different purpose for a common goal.

Among our secondary outcomes, we found that older age and male sex were associated with worse outcome. This was shown by previous observational studies, too (Kartsonaki et al., 2023). Possible explanations might be: differences in health behaviors, as men are more prone to smoke and alcohol consumption than women, higher prevalence of hypertension and cardiovascular diseases in men generally, as well as increased prevalence of obesity, diabetes, hypertension, and cardiovascular diseases in elderly men (Shim et al., 2020). Previously, also obesity and other comorbidities (in particular hypertension and type 2 diabetes) have been associated with increased risk of severe disease and death (Kartsonaki et al., 2023), but in our study they were not.

The lack of detailed information about the specific comorbidities and the body mass index (the variable was ‘obesity’, with no gradient) are among the limitations of this study. Another limitation is due to the use of drug classes as a whole (i.e., NSAIDs, steroids, antibiotics). Finally, despite the large number of patients’ records available in the unCoVer platform, we could use only data from two sites, which reported the main variable of interest. However, this study shows the potential of federated analyses, which can be done taking advantage of a large harmonized database of real-world data provided by multiple sites.

## 5 Conclusion

Taken altogether with previous evidence, the domiciliary use of NSAIDs seems to be appropriate to treat symptoms due to SARS-CoV-2 infection, with no increased cardiovascular risk found in association with this drug class in these patients. Concerns about a benefit in terms of reduced hospitalization persist, and here we add evidence showing that their use might not impact in-hospital mortality. In conclusion, domiciliary NSAIDs use would not be recommended *per se* in case of SARS-CoV-2 infection, that is in the absence of symptoms for which the patient would benefit from this treatment. Finally, neither other drugs (steroids, antibiotics) nor comorbidities influenced the clinical outcome in our analysis. An increased risk of death was instead associated with increasing age and male sex.

## Data Availability

All data are available in the inCoVer platform: https://uncover-eu.net/data/.
